# *S. aureus* Biofilm Protein Expression Linked to Antimicrobial Resistance: A Proteomic Study

**DOI:** 10.3390/ani11040966

**Published:** 2021-03-31

**Authors:** Cristian Piras, Pierluigi Aldo Di Ciccio, Alessio Soggiu, Viviana Greco, Bruno Tilocca, Nicola Costanzo, Carlotta Ceniti, Andrea Urbani, Luigi Bonizzi, Adriana Ianieri, Paola Roncada

**Affiliations:** 1 Department of Health Sciences, University “Magna Græcia” of Catanzaro, Campus Universitario “S. Venuta”, Viale Europa, I-88100 Catanzaro, Italy; c.piras@unicz.it (C.P.); tilocca@unicz.it (B.T.); costanzo.nic@unicz.it (N.C.); ceniti@unicz.it (C.C.); 2 Department of Veterinary Sciences, University of Torino, Largo Paolo Braccini 2, Grugliasco, 10095 Torino, Italy; pierluigialdo.diciccio@unito.it; 3 Surgical and Dental Sciences-One Health Unit, Department of Biomedical, University of Milano, Via Celoria 10, 20133 Milano, Italy; alessio.soggiu@unimi.it (A.S.); luigi.bonizzi@unimi.it (L.B.); 4 Department of Basic Biotechnological Sciences, Intensivological and Perioperative Clinics, Università Cattolica del Sacro Cuore, Largo Francesco Vito 1, 00168 Roma, Italy; viviana.greco@unicatt.it (V.G.); andrea.urbani@unicatt.it (A.U.); 5 Molecular and Genomic Diagnostics Unit, Fondazione Policlinico Universitario Agostino Gemelli IRCCS, Largo A. Gemelli 8, 00168 Roma, Italy; 6 Deparment of Food and Drug, University of Parma, Parco Area delle Scienze 27A, 43124 Parma, Italy; adriana.ianieri@unipr.it

**Keywords:** *Staphylococcus aureus*, planktonic cells, biofilm, proteomics, food safety, antimicrobial resistance

## Abstract

**Simple Summary:**

Biofilm formation represents one of the most effective forms of bacterial persistence in surfaces where nutrients are available or in the tissues of living hosts as humans or animals. Such persistence is due to the high rate of antimicrobial resistance of this shell conformation. It often represents a burden when the pathogen colonizes niches from where it is not removable such as food facilities, farm facilities or parts of living organisms. In this study, we investigated biofilm formation mechanisms and enhanced antimicrobial resistance of 6 different *S. aureus* strains. The detected mechanisms were primarily related to the control of catabolites, the production of proteins with moonlighting activities and the detoxification of compounds with antimicrobial activities (i.e., alcohol). Glycolysis and aerobic metabolisms were found to be less active in the biofilm conformation. Consequently, less H_2_O_2_ production from aerobic metabolism was translated into a measurable under-representation of catalase protein.

**Abstract:**

Antimicrobial resistance (AMR) represents one of the most critical challenges that humanity will face in the following years. In this context, a “One Health” approach with an integrated multidisciplinary effort involving humans, animals and their surrounding environment is needed to tackle the spread of AMR. One of the most common ways for bacteria to live is to adhere to surfaces and form biofilms. *Staphylococcus aureus* (*S. aureus*) can form biofilm on most surfaces and in a wide heterogeneity of environmental conditions. The biofilm guarantees the survival of the *S. aureus* in harsh environmental conditions and represents an issue for the food industry and animal production. The identification and characterization of biofilm-related proteins may provide interesting insights into biofilm formation mechanisms in *S. aureus*. In this regard, the aims of this study were: (i) to use proteomics to compare proteomes of *S. aureus* growing in planktonic and biofilm forms in order to investigate the common features of biofilm formation properties of different strains; (ii) to identify specific biofilm mechanisms that may be involved in AMR. The proteomic analysis showed 14 differentially expressed proteins among biofilm and planktonic forms of *S. aureus*. Moreover, three proteins, such as alcohol dehydrogenase, ATP-dependent 6-phosphofructokinase, and fructose-bisphosphate aldolase, were only differentially expressed in strains classified as high biofilm producers. Differentially regulated catabolites metabolisms and the switch to lower oxygen-related metabolisms were related to the sessile conformation analyzed.

## 1. Introduction

Humanity is already facing a challenge related to antimicrobial resistance (AMR). Such a burden will become worse due to the massive use of antimicrobials such as alcohol-based products for hands and workplaces sanitization necessary to mitigate the transmission of coronavirus disease 2019 (COVID-19). These key precautions may create an ecological pressure on microorganisms and contribute to the emergence of AMR in microbial populations that can colonize human body and the environment.

The use of biocides in the environment (such as farms and food industries) has already created a phenomenon known as AMR cross-resistance [1,2,3]. Biofilm formation contributes to enhance AMR resistance by physical and biochemical means [4]. A biofilm is defined as “a microbially derived sessile community characterized by cells that are irreversibly attached to a substrate or interface or to each other, are embedded in an auto-produced matrix of extracellular polymeric substances (which is composed of protein, DNA and polysaccharide) and exhibit an altered phenotype with respect to growth rate and gene transcription” [5]. It is well known that bacteria growing as biofilms might be physiologically distinct from the same bacteria growing as free-swimming planktonic cells [6,7].

Briefly, biofilms allow bacteria to better resist harsh environmental conditions [8]. Such a conformation can be found everywhere where there is a source of nutrients such as in the food-processing environment or zootechnical industry (food-processing equipment, milk collection and storage facilities) [9]. Biofilms-enhanced resistance to disinfectants/antimicrobials/antibiotics represents a threat for food industries and farms [10]. The biofilm, in fact, protects the bacteria from detaching by cleaning agents and from being killed by disinfectants [11]. However, biofilm protection mechanisms appear to be different from those responsible for resistance to conventional antibiotics [12]. First, the extracellular polymeric substances (EPS) matrix delays or prevents antimicrobial action, either by limiting disinfectants diffusion or by chemical interaction/inactivation with proteins and extracellular polysaccharides [13]. Other factors can play a role in this feature, such as the bacterial growth rate, the heterogeneity within the biofilm, the general stress response, quorum sensing mechanisms, the induction of a certain biofilm phenotype and the over-expression of efflux pumps [14]. In addition, biofilm activities include the upregulation of virulence factors and secretion of extracellular polymers [15]. Horizontal gene transfer plays an important role in AMR. The small intra-cellular distance typical of biofilms facilitates the spread of resistance genes and generates the presence of extracellular DNA in the biofilm matrix [16].

Among bacteria, *Staphylococcus aureus* (*S. aureus*) is able to form biofilm on most surfaces and under almost all the environmental conditions found in food industries [17]. It is a commensal and opportunistic pathogen and under certain conditions, may cause a wide range of infectious diseases such as skin infections, bacteremia, endocarditis, pneumonia and food poisoning. *S. aureus* biofilm mode of growth is regulated by complex genetic factors and can produce at least two different types of biofilm: ica operon-dependent (i.e., promoted by the ica operon) and ica operon-independent [17]. A study carried out by Resch et al. (2005) identified more than 160 genes that were significantly over-expressed during biofilm growth conditions. Those genes encoded for binding factors, polysaccharide intracellular adhesion (PIA) and peptidoglycan modeling factors [7]. Additionally, many proteins have been implicated as important components in cellular adhesion and biofilm matrix development [18]. These include surface-associated proteins (protein A), fibrinogen-binding proteins (FnBPA and FnBPB), biofilm-associated protein (Bap) and clumping factor B (ClfB).

Considering the concerns for food safety associated with *S. aureus* biofilms and the high cost of managing this issue in the food industry, a better knowledge of the mechanisms involved in *S. aureus* biofilm growth mode is essential. To date, several studies have focused on pathogenicity and only a few have addressed differences in protein expression of *S. aureus* due to biofilm formation [19,20]. The identification and characterization of proteins linked with biofilm could provide interesting insights on the mechanism and/or process of biofilm formation in *S. aureus*.

According to this premise, the aims of this study were: (i) to compare proteomes of *S. aureus* growing in planktonic and biofilm forms, in order to investigate the common features of biofilm formation properties of six different strains; and (ii) to identify possible biofilm mechanisms that may be involved in AMR. The employment of 6 different strains will help with the comprehension of biofilm formation mechanisms more representative of the *S. aureus* species rather than be focused on mechanisms typical of a single strain.

## 2. Materials and Methods

### 2.1. Bacterial Strains

A total of six biofilm-forming *S. aureus* strains were analyzed in this study. In details, three *S. aureus* reference strains (ATCC 35556, ATCC 12600, ATCC 29213) and three food-related isolates (wild-types) were used in the experiment. The food related-strains were isolated from food (n.1) and food handlers (n.2), respectively.

Stock cultures were stored at −80 °C. All strains were incubated for 24 h at 37 °C in tryptone soy broth (TSB, Oxoid S.p.A., Milan, Italy) before each experiment. All these strains have been grown both in the planktonic and in the sessile form (biofilm cultures) and analyzed through 2D electrophoresis coupled with matrix-assisted laser desorption/ionization time-of-flight mass spectrometry (MALDI-TOF MS).

The sessile (biofilm cultures) and planktonic cells were prepared as follows:

- Biofilm cultures

The ability of *S. aureus* isolates to produce biofilms was determined according to the protocol described by Di Ciccio et al., 2015 [21]. In all cases, all experiments were repeated in triplicate. Briefly, polystyrene tissue culture plates (6 wells—961 mm^2^) were used as substratum for biofilm formation at 37 °C. Cultures of *S. aureus* were prepared, from overnight tryptone soy agar (TSA, Oxoid S.p.A., Milan, Italy) growth, in TSB by incubating at selected temperature: 37 °C. Cultures were then washed three times with sterile phosphate-buffered saline (PBS; pH 7.3) (Sigma-Aldrich S.r.l., Milan, Italy) and diluted with fresh TSB to reach a concentration of about 10^8^ colony-forming units (CFU) mL^−1^ by reading the optical density (OD) level at 550 nm (UV Mini-1240—Shimadzu, Long Beach, CA, USA). Three milliliters (ml) of the standardized inoculum were then added to polystyrene tissue culture plates (well—35 mm diameter). Samples were then incubated at 37 °C. After 24 h incubation, non-adherent cells were removed by washing each well three times with sterile PBS. After adding sterile PBS (3 mL), biofilm in wells was dislodged mechanically by scraping vigorously using a sterile cell-scraper. Finally, the cells were harvested by centrifugation (4000 rpm, 10 min., 4 °C, Beckman, J2-MC, centrifuge). The resulting pellets, washed and resuspended in sterile PBS, were centrifuged again (4000 rpm, 10 min., 4 °C). The cells were washed several times and pelleted by five centrifugations. Finally, the supernatant was removed and the pellet from the biofilm cultures grown was stored at −80 °C until use for proteomic studies (the pellets from the biofilm cultures had a weight of 50 mg).

- Planktonic cells

*S. aureus* reference strains (ATCC 35556, ATCC 12600, ATCC 29213) and food-related isolates *S. aureus* (281, 402, 184) were used. An overnight culture was created by inoculating a colony of *S. aureus* into 5 mL of TSB for 24 h at 37 °C. After incubation, the *S. aureus* culture was centrifuged for 10 min at 4000 rpm, 4 °C. The supernatant was then replaced with sterile PBS, and pellet was resuspended by thoroughly mixing with pipette. The cells were washed several times and pelleted by five centrifugations (4000 rpm, 10 min, 4 °C). Finally, the supernatant was removed and the pellet from the overnight cultures grown was stored at −80 °C until use for proteomic studies (the pellets from the planktonic cultures had weights: 50 mg).

### 2.2. Proteomic Analysis

- Protein Extraction and 2-Dimensional Electrophoresis (2-DE)

We diluted 50 milligrams of cellular pellet of the different *S. aureus* strains in 700 µL of 2DE buffer containing 7 M urea, 2 M thiourea, 4% CHAPS, 1% DTT, and protease inhibitors (GE-Healthcare) according to manufacturer instructions.

To ensure the complete disruption of the collected bacterial cells, the samples were processed with 6 cycles of 1-min bead beating interspersed by a cycle of centrifuge. For this purpose, into the sample was added the same amount (1:1 *v/w*) of 0.1 mm zyrcounium-silica beads (300 µg beads added to 300 µL of buffer + the volume of the pellet). The bead beating cycle was conducted at 4000 rpm for 1 min with the purpose to avoid overheating. Then, the samples were centrifuged at 12,000× *g* for 5 min at 4 °C in order to chill and disperse the foam. This operation was repeated 6 times. After the 6th cycle, samples were centrifuged for 20 min and the supernatant was stored in another tube for subsequent proteomics analysis.

Two-dimensional (2D) electrophoresis was run in all samples: 100 micrograms of protein were loaded on a 7 cm strip through active rehydration performed overnight at 50 V in a buffer containing 7 M urea, 2 M thiourea, 2% CHAPS, 0.5% ampholytes 3–10 Amersham, and 26 mM DTT. For isoelectric focusing (IEF), the following protocol was applied: 100 V/1 h linear, 250 V/2 h linear, 4000 V/5 h linear, 4000 V step/50,000 total volt-hours (VhT), using a protean IEF platform.

Once the final amount of VhT was reached, immobilized pH gradient (IPG) strips were frozen up to the next step or directly equilibrated in two steps of 15 min under gentle stirring. The first step of equilibration was performed in buffer (6 M UREA, 2% SDS, 0.05 M Tris-HCl pH 8.8, 20% glycerol) supplemented with 1% DTT *w/v* and the second step was performed in a buffer with the addition of 2.5% *w/v* iodoacetamide. The IPG strips were put in a 12% home-made acrylamide gel and IEF run under constant amperage of 15 mA per gel, until the bromophenol blue (BFB) reached the front. The gels were then eliminated from the plates, washed three times with double-distilled water and spotted overnight (ON) with Coomassie Brilliant Blue.

Using an Imagescanner III (GE Healthcare) the gels were digitalized. The image analysis was performed using SameSpots software (Version 4.5, Nonlinear Dynamics U.K.). All imported images were checked for quality (saturation, ending) and spots, with a *p*-value lower than 0.05, were manually excised for subsequent mass spectrometry (MS) analysis and protein identification. If the MALDI MS/MS identification was obtained with a MASCOT score higher than 40, the protein was analyzed via GO for the comprehension of its function/role.

- Protein Identification by Matrix-Assisted Laser Desorption/Ionization Time-of-Flight Mass Spectrometry (MALDI-TOF/TOF MS) Analysis

Protein identification was performed according to previous studies [22,23].

Briefly, after different steps of dehydration, reduction and alkylation, the excided single spots were digested with a solution of 0.01 μg/μL of porcine trypsin (Promega, Madison, WI, USA) at 37 °C o.n., and peptides were concentrated using C18 ZipTip (Millipore, Bedford, MA, USA). they were then co-crystallized with a solution of αciano-4-hydroxycinnamic acid and spotted on a Ground Steel plate (Bruker-Daltonics, Bremen, Germany).

The MS analysis was performed on a Ultraflex III MALDI-TOF/TOF spectrometer (Bruker-Daltonics) in positive reflectron mode.

External calibration was performed using the standard peptide mixture calibration (m/z: 1046.5418, 1296.6848, 1347.7354, 1619.8223, 2093.0862, 2465.1983, 3147.4710; Bruker-Daltonics).

FlexAnalysis 3.3 software (Bruker-Daltonics) was used for the selection of the monoisotopic peptide masses of each mass spectra. After an internal calibration on autolysis peaks of porcine trypsin (m/z: 842.509 and 2211.104) and exclusion of contaminant ions (known matrix and human keratin peaks), the created peak lists were analyzed by MASCOT v.2.4.1 algorithm (www.matrixscience.com, accessed on 23 March 2021) searching against SwissProt 2021_database restricted to Firmicutes and *Staphylococcus aureus* (11,196 sequences) taxonomy.

The parameters used for database search are the following: carbamidomethylation of cysteines and oxidation on methionine as fixed and variable modifications respectively; one missed cleavage site allowed for trypsin; 70 ppm as maximal tolerance.

Mascot protein scores greater than 50 were considered significant (*p* < 0.05) for protein identification assignment.

To confirm the identifications, MS/MS spectra were also acquired by switching the instrument in LIFT mode with 4–8 × 10^3^ laser shots. For the fragmentation, precursor ions were manually selected, and the precursor mass window was automatically set. Spectra baseline subtraction, smoothing (Savitsky–Golay) and centroiding were operated using Flex-Analysis 3.3 software.

The parameters used for the database search are the following: carbamidomethylation of cysteines and oxidation on methionine as fixed and variable modifications respectively; one missed cleavage; 50 ppm and 0. 5 Da as mass tolerance for precursor ions and for fragments respectively. The taxonomy was restricted to *Staphylococcus aureus* (10,227 sequences).

The confidence interval for protein identification was set to 95% (*p* < 0.05) and only peptides with an individual ion score above the identity threshold were considered correctly identified.

## 3. Results

The proteomic analysis was performed in order to discover the mechanisms of biofilm formation common to all analyzed *S. aureus* strains. Six different strains with different biofilm formation indexes were analyzed in the planktonic form and the biofilm form. For each strain, biofilm formation, expressed as BPI, was calculated as follows: “BPI = [OD_mean biofilm_ surface (mm^2^)^−1^] × 1000”. All isolates were defined in different categories (weak, moderate or strong producers) on the basis of their BPIs values (Table 1).

The analyzed strains included: *S. aureus* ATCC 35556, already described as a strong biofilm producer [24,25]; *S. aureus* ATCC 12600, classified as moderate biofilm producer [21]; three food isolates of *S. aureus* classified as strong (281), moderate (402) and weak biofilm producer (184); *S. aureus* ATCC 29213 measured as weak biofilm producer. BPI on polystyrene at 37 °C was used as the measure for all the experimental procedures in this work. All the strains with BPI below 0.300 were considered weak biofilm producers. In these cases, the biofilm layer was phenotypically barely visible and not stable in its structure. Such a phenotype was confirmed by the extremely low BPI below 300. For this reason, four strains (A, B, C and D) were considered as part of the moderate/high biofilm-producing group, while the remaining two (E and F) showed a phenotype closer to the low/non-forming biofilm group.

Proteomics analysis was carried out to compare the sessile versus the planktonic phenotype; however, a separated analysis was performed, including only the moderated to strong biofilm producers. The differentially represented proteins were chosen according to the Progenesis SameSpots provided analysis of variance (ANOVA) *p*-value. The topmost significant ones were chosen to be analyzed via MALDI-TOF MS/MS peptide mass fingerprinting (PMF) and peptide fragment fingerprinting (PFF) if necessary. Of the chosen spots, only the ones successfully identified with a MASCOT score higher than 40 were considered for subsequent Gene Ontology (GO), metabolism and pathway analysis.

As reported in Table 2, 14 proteins were differentially expressed among *S. aureus* planktonic and sessile groups. Of these, 11 were differentially expressed when considering all the strains together with a *p*-value ≤ 0.05 (column: regulation in planktonic vs. sessile). Alcohol dehydrogenase, ATP-dependent 6-phosphofructokinase and Fructose-bisphosphate aldolase differential expression were significant for the medium/high biofilm-forming sub-group (high biofilm producers’ column). This classification was done according to the observation of the datasets that clearly showed how the representation trend of some of the differentially expressed proteins was clearly not following the same path in the weak biofilm forming strains. As previously mentioned, this was the case for alcohol dehydrogenase, ATP-dependent 6-phosphofructokinase and fructose-bisphosphate aldolase.

If considering the entirety of the differentially regulated proteins, five were found to be over-represented in the sessile versus planktonic group, and 9 proteins were found to be under-represented. This low number of detected proteins might be due to the high heterogeneity of the different strain analyzed. Three of the five over-represented proteins were involved in carbon metabolism or in stress response. Interestingly, alcohol dehydrogenase and 30 s ribosomal proteins are involved in antimicrobials resistance mechanisms, i.e., detoxification.

On the other hand, under-represented proteins such as 2,3-bisphosphoglycerate-dependent phosphoglycerate mutase, alkyl hydroperoxide reductase subunit C, ATP-dependent 6-phosphofructokinase, catalase etc. were mostly involved in energy and oxygen-related metabolism.

**Figure 1:**
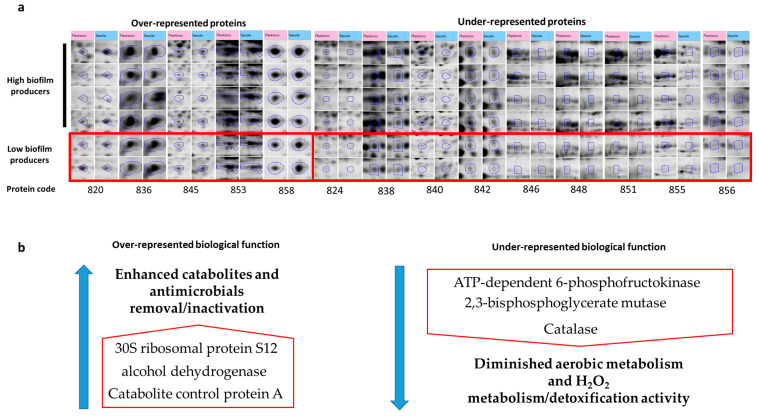
(**a**) Graphic representation of the differentially expressed proteins mostly relevant to the regulation of the described mechanisms/pathways. (**b**) Representation of the differentially regulated proteins and the related modulated mechanisms.

All data are shown in Table 2 and the image of the differentially represented proteins is shown in Figure 1a. For each protein, it is represented the related figure from the image analysis software. Table 2 indicates the *p*-values obtained from the built-in ANOVA test of the Progenesis SameSpots software. For each protein it is provided with the UNIPROT name and accession number (first two columns of the table); the SameSpots coding number, which represents the code provided by the image analysis software; the Mascot score identification obtained by the combined MALDI peptide mass fingerprint together with the peptide fragment fingerprint for the MS/MS identification; the number of matched peptides and the mascot score; and the ANOVA *p*-value obtained by comparing the planktonic and sessile form of all strains and just moderate/high biofilm producers (last column, the values of normalized volume for each spot are provided in Supplementary Materials, Table S1).

Figure 1a provides a graphic representation of the Coomassie Brilliant Blue stained entire proteins as detected by the image analysis software. The top four rows show high and moderate biofilm producers’ spots, while the two rows at the bottom indicate the low biofilm producers.

Figure 1b shows the graphic representation of the most relevant differentially regulated proteins and metabolisms among the two analyzed *S. aureus* phenotypes. Biological functions were manually checked after each GO search and subsequently reported in the scheme in Figure 1b.

## 4. Discussion

Biofilms growth is the preferred strategy for the expansion and survival of many clinically and environmentally relevant microorganisms [5]. *S. aureus* is one well-known biofilm-forming pathogen capable of colonizing medical devices [26], food contact surfaces [21] and farm industry facilities [27]. In the biofilm form, *S. aureus* can successfully cope against strong stress conditions [28] and persist on the surfaces of food-processing plants [4], leading to recurrent contamination of both fresh and processed foods worldwide [29,30,31,32]. From this perspective, biofilm formation represents a severe threat because of its difficult removal linked to the extremely high tolerance to antimicrobials and antibiotics. Improving knowledge about its formation mechanisms and pathways is mandatory to better design possible and practical intervention strategies. Studies performed on single strains (strain-specific mechanisms) documented the over-representation of fibrinogen-binding protein and the accumulation-associated protein (Aap) in *S. aureus* cells growing embedded in the biofilm matrix in comparison to those growing in the planktonic form [33,34]. Also, increased production of staphylococcal accessory regulator A (SarA) was shown in biofilm formation [20].

All these and many other studies extensively describe the physiology of *S. aureus* biofilm formation that is specific to the strain analyzed. However, it is not considered that diverse *S. aureus* strains may have different mechanisms and pathways of biofilm formation.

In the current study, we employed a comparative proteomic approach to understand better the process of biofilm formation and the possible mechanisms involved in the enhancement of antimicrobial resistance. To achieve this result, we performed a differential proteomics analysis of planktonic versus sessile *S. aureus* isolates and ATCC strains. Six different strains with a wide range of biofilm formation indexes were employed in order to maximize the possibility to detect general mechanisms more representative of the *S. aureus* specie.

The whole comparison allowed the discovery of 14 proteins differentially regulated between the planktonic and sessile group and, three of those (alcohol dehydrogenase, ATP-dependent 6-phosphofructokinase and fructose-bisphosphate aldolase) were specific to the high biofilm-producing strains.

Ribosomal proteins are involved in biofilm regulation/formation and enhanced antimicrobial resistance [35,36]. Interestingly, changes at the ribosomal protein isoforms can shape the response to antibiotics by modifying the affinity of tetracyclines, chloramphenicol, macrolides (e.g., erythromycin) and aminoglycosides (e.g., streptomycin) for the transcription machinery. Hence, a switch in the composition of ribosomal subunits could be involved in biofilm formation and the different susceptibility to antimicrobial molecules [37].

Fructose bisphosphate aldolase and catabolite control protein A (ccpA) are over-represented in the biofilm conformation versus the sessile condition. The first is an essential enzyme of the glycolytic pathway with virulence functions shaped according to its cellular localization (i.e., moonlighting properties) [38]. As a moonlight protein, it is often expressed in the bacterial surface [39] where it has been linked to virulence in several bacterial pathogens, such as *Francisella* [40], by directly affecting cell migration through its interference with the actin polymerization process.

Similarly, fructose bisphosphate aldolase expression is induced in oxygen depletion conditions, and it has also been associated with transcriptional regulator functions [39]. Catabolite control protein A (ccpA) was found to be massively over-represented in both high and low biofilm producers growing in the sessile conditions. This might be explained by the requirements of the typical multi-layered packed structure of the biofilm, which needs a tight control of nutrients availability, catabolites and secondary metabolites (e.g., ethanol, reactive oxygen species (ROS) etc.). Indeed, nutrients depletion or catabolites accumulation would exert toxic/detrimental effects on the bacterial community itself. In Gram+ bacteria, ccpA expression regulates the synthesis of capsular polysaccharides, toxigenic exoproteins and promotes biofilm formation [25]. Similarly, *S. epidermidis* biofilm formation is positively regulated by ccpA and causes tricarboxylic acid (TCA) cycle repression [41]. This demonstrates that the management of carbon and energy flow by regulating the enzymes involved in glycolytic/fermentative metabolism [42] represents an essential element for the proper formation of biofilm. Accordingly, previous evidence reported that environmental acidification or other phenomena associated with rapid metabolism of carbohydrates occurring in bacteria growing in sessile conditions are regulated by ccpA throughout the modulation of pfka and gpma expression [42,43]. Moreover, the structural organization of the biofilm is likely to result in the accumulation of toxic secondary metabolites such as ethanol from fermentation processes. This may explain the detected increased expression of alcohol dehydrogenase (adh) in the sessile growing strains. The oxygen depletion in the biofilm’s inner layers may cause a metabolic shift towards the mixed alcoholic fermentation with increased ethanol concentration that needs to be promptly detoxified by the induction of adh [44,45,46]. The hypothesis of the metabolic shift towards fermentation and ethanol production is also supported by the under-expression of PfkA and gpmA, which are active in pyruvate production. By limiting the production of pyruvate, sessile cells control the pathways towards any possible fermentative process. Thus, the reduced abundance of PfkA and gpmA in the sessile bacteria might represent the effect of a negative feedback modulation of the fermentative process to protect the bacteria from the toxicity of their secondary metabolites. Analogously, the reduced abundance of catalase, the enzyme active in ROS detoxification, may be a consequence of the reduced oxygen availability in the bacterial samples growing in biofilm form [47]. Such a reduction in the hydroperoxide detoxification power is confirmed by the down-regulation of 3 different catalase isoforms and of alkyl hydroperoxide reductase subunit C (Q2FJN4). This may help to explain the high power of low doses of hydrogen peroxide to dissolve the biofilm conformation [48].

## 5. Conclusions

The comparative top-down proteomics (2D-electrophoresis–MALDI TOF) approach used here identified some possible biofilm formation mechanisms of *S. aureus* strains with a wide range of biofilm formation indexes. Biofilm is one of the essential strategies for bacterial virulence and persistence over a wide variety of surfaces and unfavourable conditions and it facilitates survival and resistance in the presence of antimicrobial compounds [49]. Comparison of high- and low-biofilm forming strains in sessile and planktonic form highlighted common mechanisms as the catabolite control and the modulation of the detoxification machinery aimed at avoiding self-inhibition/toxicity (i.e., ethanol detoxification). Glycolysis and aerobic metabolisms seem to be down-regulated in favor of possible fermentation pathways that might be responsible for ethanol production and, possibly, for the induction of alcohol dehydrogenase production.

This study is characterized by using a top-down proteomics approach that led the differential quantification of intact proteofoms. On the other hand, this approach limits the detection of differentially represented, less-abundant proteins. Complementing these data with shotgun proteomics and metabolomics is desired to support the observed evidence and to discover potential biomolecular targets to contrast and/or attenuate this phenomenon.

## Figures and Tables

**Table 1:** Biofilm formation index (BPI) of *S. aureus* strains on polystyrene at 37° included in this study.

Strains	Source	BPI
A—*S. aureus* ATCC n.35556	ATCC n.35556	0.758
B—*S. aureus* ATCC n.12600	ATCC n.12600	0.405
C—*S. aureus* n.281	Food	1.019
D—*S. aureus* n.402	Food-handler	0.311
E—*S. aureus* n.184	Food-handler	0.290
F—*S. aureus* ATCC 29213	ATCC n.29213	0.260

**Table 2:** List of differentially represented proteins among the six different strains analyzed under planktonic and biofilm conditions. As in the last two columns, the analysis was performed, including all the analyzed strains and, subsequently, excluding the low biofilm producers (last column). OS= organism name. Every significant *p*-value (lower than 0.05) is printed in bold.

Uniprot Name/Accession Number	Uniprot Name	SameSpots Coding Number	Protein Name	Mascot Score	Sequence Coverage (%)	N of Matched Peptides	Regulation in Sessile vs. Planktonic	High Biofilm Producers (Sessile vs. Planktonic)
Q2FJ31	ADH_STAA3	820	alcohol dehydrogenase	66	35	9/59	↑0.070	**↑0.018**
A7X656	GPMA_STAA1	824	2,3-bisphosphoglycerate-dependent phosphoglycerate mutase OS = *Staphylococcus aureus*	116	51	11/52	**↓0.001**	**↓0.016**
P0A0H0	RS12_STAA8	836	30S ribosomal protein S12	68	37	4/45	**↑0.026**	0.135
Q2FJN4	AHPC_STAA3	838	Alkyl hydroperoxide reductase subunit C OS = *Staphylococcus aureus*	67	39	6/31	**↓0.028**	0.137
A6U2G5	PFKA_STAA2	840	ATP-dependent 6-phosphofructokinase OS = *Staphylococcus aureus*	121	35	11/31	↓0.084	**↓0.007**
A7WZR9	CLPP_STAA1	842	ATP-dependent Clp protease proteolytic subunit OS = *Staphylococcus aureus*	76	30	9/23	**↓0.008**	0.084
Q5HF38	CCPA_STAAC	845	Catabolite control protein A OS = *Staphylococcus aureus*	76	26	9/35	**↑0.001**	**↑0.004**
Q9L4S1	CATA_STAAU	846	Catalase OS = *Staphylococcus aureus* GN = katA PE = 3 SV = 1	40	16		**↓0.041**	**↓0.041**
Q9L4S1	CATA_STAAU	848	Catalase OS = *Staphylococcus aureus* GN = katA PE = 3 SV = 1	68	30	6/40	**↓0.021**	**↓0.027**
Q9L4S1	CATA_STAAU	851	Catalase OS = *Staphylococcus aureus* GN = katA PE = 3 SV = 1	98	47	11/40	**↓0.012**	**↓0.004**
Q2FDQ4	ALF1_STAA3	853	Fructose-bisphosphate aldolase class 1 OS = *Staphylococcus aureus*	112	40	17/67	↑0.096	**↑0.0002**
Q2YSZ4	GCSPB_STAAB	855	Probable glycine dehydrogenase (decarboxylating) subunit 2 OS = *Staphylococcus aureus*	68	26	9/50	**↓0.037**	**↓0.013**
A7X395	ENGB_STAA1	856	Probable GTP-binding protein EngB OS = *Staphylococcus aureus* _12042016_	68	16	4/16	**↓0.007**	0.060
Q7A551	Y1532_STAAN	858	putative universal stress protein SA153 (782) OS = *Staphylococcus aureus*	86	36	6/35	**↑0.010**	0.094

## Data Availability

Not applicable.

## Supplementary Materials

The following are available online at https://www.mdpi.com/article/10.3390/ani11040966/s1, Table S1: Raw normalized volume.
